# Announcement: Third Keele Meeting on Aluminium the Bioinorganic Chemistry of Aluminium: From Microbe to Man

**DOI:** 10.1155/MBD.1998.315

**Published:** 1998


### THIRD KEELE MEETING ON ALUMINIUM
### The Bioinorganic Chemistry of Aluminium: from Microbe to Man

### 22nd-24th February 1999

### Birchall Centre for Inorganic Chemistry and Materials Science Keele University

**Aims of the Meeting**

Aluminium is one of only a few elements to have been discussed at all levels of public life. Unfortunately, much of the debate concerning its impact upon biota, including man, has been uninformed and has served only to trivialise this important field of research. The study of aluminium and its activity in biological systems is easily justified. Its ubiquity alone suggests biological function, and yet, none has been found. What is clear is that aluminium is found in all biota and that this occurrence is either the vestige of a metal that has been selected out of biological processes or the result of a burgeoning exposure which is currently an active player in natural selection. It is, perhaps, the latter possibility that courts such red mists of controversy? Our role as scientists is to look beyond the controversy and to provide the best possible indications of the biological activity of aluminium. All researchers working on aluminium will contribute to this process. The Keele Meetings promote a multidisciplinary approach bringing together all scientists with an interest in the chemistry and biology of aluminium. The term, bioinorganic chemistry, is simply a description of how research from different fields can be brought together and, perhaps, re-interpreted to offer an interdisciplinary explanation of the observed effects of biologically available aluminium. The continued aim of the Keele Meetings on Aluminium is to promote such an approach.

**Call for Papers**

The Keele Meetings provide a forum for the presentation of new, previously unpublished research relating to the field of Aluminium in Biology. Papers are also invited on the related topics of Silicon Essentiality and Silicification in Biota. Platform Presentations should be of 20 minutes duration to allow a further 10 minutes for discussion. Participants presenting Posters will be offered a 5 minute slot within a related Platform Session to "advertise" their Poster. A prize will be presented for the Best Poster.

**J. D. Birchall Memorial Lecture**

In 1999 the J. D. Birchall Memorial Lecture will be given by Professor R. J. P. Williams FRS of the University of Oxlord.

**Preliminary Timetable**

*Monday 22nd February 1999*
11.00 Registration
12.30 Lunch
14.00 Conference
18.00 Poster Presentations
19.30 End o 1st Day

*Tuesday 23rd February 1999*
09.00 Conference
12.30 Lunch
14.00 Conference
18.00 End of 2nd Day
20 00 Conference Dinner

*Wednesday 24th February 1999*
09.00 Conference
12.30 Lunch
13.30 J. D. Birchall Memorial Lecture
14.30 Discussion
15.30 End of Conference

Details of talks and speakers will be finalised and posted by December 1st 1998.

**Registration**

The Conference is run at cost. All participants will pay the Conference Fee of £ 230.00 per person This includes registration, accommodation, all meals (including the Conference Dinner) and the use of all of the Hotel Facilities.

Delegates wishing to share a twin or double room will be charged at £ 200.00 per person.

Delegates not requiring accommodation will be charged £150.00.

Delegates requiring accommodation at the Hotel before or after the meeting should contact Dr. C. Exley for information.

A small number of postgraduate bursaries may lbe available for students making presentations. Contact Dr. C. Exley for more information.

To help to maintain the preferred informal atmosphere of the meeting the number of delegates will be strictly limited to 120. Pease confirm your intention to attend the meeting as soon as possible. Please ensure that the Conference Fee is paid by 31 st December 1998.

Payment should be by cheque, in sterling, made payable to Keele Utliversity and sent to the address below

Dr. C. Exley
Birchall Centre for Inorganic Chemistry and Materials Science
Department of Chemistry, Keele University
Staffordshire ST5 513G, UK

Inquiries to

Dr. Chris Exley Tel: 01782 584080 Fax 01782 715944 Email: cha38@keele.ac.uk

Following the acknowledged successes of the first two Keele Meetings we have decided not to break with tradition and to hold the Third Meeting at the same venue, the Stakis Hotel in Hanley, Stoke-on-Trent. The Hotel is situated in the heart of the city of Stoke-on-Trent and is conveniently located for both rail and road travel. Car parking is available at the Hotel and delegates will have use of all the hotel facilities including the swimming pool.

The Stakis Hotel, Trinity Street, Hanley
Stoke-on-Trent ST1 5NB
Tel: 01782 202361

